# Harmonizing scientific rigor with political urgency: policy learnings for identifying accelerators for scale-up from the safe childbirth checklist programme in Rajasthan, India

**DOI:** 10.1186/s12913-019-4093-2

**Published:** 2019-05-02

**Authors:** Somesh Kumar, Priti Dave, Ashish Srivastava, Jelle Stekelenburg, Dinesh Baswal, Deepti Singh, Bulbul Sood, Vikas Yadav

**Affiliations:** 1 Jhpiego-an affiliate of Johns Hopkins University, 29, Okhla Phase -3, New Delhi, 110019 India; 2 Department of Health Sciences, Global Health, University Medical Centre Groningen, University of Groningen, Groningen, the Netherlands; 3 Children’s Investment Fund Foundation (CIFF), London, United Kingdom; 4 Department of Obstetrics and Gynaecology, Leeuwarden Medical Centre, Leeuwarden, the Netherlands; 5 Maternal Health Division, Ministry of Health & Family Welfare, Government of India, New Delhi, India

**Keywords:** Scale-up, Public health interventions, Low and middle income countries, Safe childbirth checklist programme, Maternal and newborn health

## Abstract

**Background:**

Quick scaling-up of innovative and promising interventions in health systems of low and middle-income countries to rapidly achieve population level benefits is a key challenge. While there is consensus on the need for rigorous scientific evidence on effectiveness of interventions before considering scale-up, there can be significant time lag for the want of gold-standard evidence. The Safe Childbirth Checklist (SCC) programme in India, demonstrated how an innovation was robustly evaluated and scaled up nationally, within a short span of time. In this narrative review, we describe the strategies discussed in various published scale-up frameworks and map them against the strategies adopted by the SCC programme to identify accelerators which facilitated its rapid scale up.

**Methods:**

The narrative review – done from May to June 2017 - involved keyword searches of electronic databases of PubMed, Ovid Medline and Google Scholar. It included the key words ‘pilot’, ‘health innovations’, ‘scale-up’, ‘replication’, ‘expansion’, ‘increased coverage’, ‘conceptual models for scale-up’, ‘frame-works for scale-up’, ‘evidence for scale-up’ in the title of publications,. This search was limited to publications in English after the year 1995. We used snowball sampling approach (by referring to bibliographies of shortlisted publications) to identify additional publications related to scale-up. We then screened the identified publications independently and relevant publications that discussed attributes for a conceptual model for scale-up of public health interventions in low and middle-income countries were shortlisted. We then mapped the strategies we used in SCC program scale up against those described in the shortlisted frameworks to identify seven accelerators which facilitated rapid scale up.

**Results:**

The identified accelerators were: testing the intervention in real world, resource constrained settings; using an appropriate and time sensitive research design; testing the intervention at substantial scale and in diverse settings; using an adaptive and iterative prototyping approach for implementation; sharing data and evidence with key stakeholders on an ongoing basis; targeting bridge resources through strategic engagement of stakeholders and timely integration of scale-up plans with annual planning and budgeting cycles and systems.

**Conclusion:**

These accelerators will complement current frameworks and provide guidance to future scale-up initiatives in India and elsewhere.

## Background

Though impressive progress was made towards achievement of the Millennium Development Goals (MDGs) in low- and middle income countries (LMIC), many missed the MDGs- 4 and 5 goals of reducing under-five child and maternal mortality [1]. A huge burden of preventable maternal and child mortality still exists in LMIC [2, 3]. For example, India still accounts for 25% of all global newborn deaths [4]. This situation has persisted over the last decade despite knowledge of interventions needed to address major causes of maternal and newborn mortality [5–7]. The inability of health systems to scale-up evidence-based interventions to achieve population-level benefits in a time sensitive manner is a challenge. This is particularly important as in many LMIC the political landscape changes rapidly thereby creating a paucity of committed resources to facilitate progression from actionable evidence to scaling up interventions. In addition, newer priorities may emerge that can draw attention or resources from interventions under test for scale-up.

A review of available literature on scale-up strategies reveals that multiple frameworks have been developed to guide the scale-up process based upon critical enabling factors which increase the chances of newly proven interventions being taken up by governments. Most of these frameworks take a sequential, structured approach starting with testing an innovation, and once proven to be effective - promoting its scale-up for increased coverage [8–11]. A general limitation of these conceptual frameworks is that they do not explicitly address the need to make rapid progress, apart from referring to the types of innovations that tend to disperse faster. This is a glaring omission, given the urgency of the problems, ethical obligations and political compulsion to act fast and make rapid progress. Need for urgency is important from an efficiency perspective, but can be at odds with the interests and priorities of the research community, which often favours ‘gold-standard’ and time intensive evaluation methods for evidence generation. The conventional scale-up frameworks also do not address how best to harmonise scientific rigour with the political and ethical urgency to rapidly reduce preventable maternal and child mortality.

WHO developed the SCC in 2009, in collaboration with Harvard University and other global experts, as a tool to help improve quality of childbirth care. It prompts maternity staff to perform 28 critical practices during admission at the health facility and during delivery, immediately after birth and before discharge of mother and the newborn [12]. In a pilot pre-post intervention study conducted in rural Karnataka, India, in 2010, it was seen that the use of SCC led to a significant improvement in provider adherence to these critical practices [13].

Though the initial study demonstrated the SCC as a promising tool, there was a need to test it at a reasonable scale and assess its impact on neonatal mortality. Jhpiego in partnership with the state Government of Rajasthan (GoR), India implemented a programme to improve the quality of intrapartum care in public sector health facilities of the state from the year 2012 to 2015. In this programme, the WHO Safe Childbirth Checklist (SCC), a tool aimed at improving health provider adherence to 28 essential practices during labour and immediately after birth [12], was tested in 100 public health facilities using a quasi-experimental evaluation approach. The implementation of the SCC programme was found to significantly improve the adherence to safe care practices in the target facilities [14] leading to an 11% reduction in stillbirths and early neonatal deaths [15]. Upon the learning from the SCC experience, the Government of India (GoI) launched a scale-up version in 8 Indian states with highest neonatal mortality rates in 2015. So far, such a rapid scale-up has not happened in any other country despite many focused pilot projects [16–19]; the availability of an SCC implementation guide by WHO, and the SCC featuring in the list of the most promising technologies of the decade [20]. The SCC programme illustrated that it is possible to prove an innovation in a robust manner and secure government commitment and readiness for national scale-up [21] within a reasonably short time span, 3 years in this case.

The aim of this paper is to study in depth the existing scale-up frameworks described for public health innovations in LMICs; ,and compare the strategies adopted in Rajasthan for rapid scale-up in India with those described in frameworks to identify key accelerators which facilitate rapid scale up. This new knowledge will complement the current frameworks and provide guidance to future scale-up initiatives in India and elsewhere.

## Methods

We conducted a narrative review. Narrative review articles describe and discuss the state of the science of a specific topic or theme from a theoretical and contextual point of view [22].

In this study, we followed two main methodological approaches.

1. We (SK, PDS, VY and AS) did a literature review to identify peer-reviewed publications and grey-literature on public health innovations scale-up. Using different Booleans of keywords and constructs - ‘pilot’, ‘health innovations’, ‘scale-up’, ‘replication’, ‘expansion’, ‘increased coverage’, ‘conceptual models for scale-up’, ‘frame-works for scale-up’, ‘evidence for scale-up’ in the title of publications, we searched the online databases of PubMed, Ovid Medline and Google Scholar. This search was conducted in May and June 2017. It was limited to publications in English after the year 1995, to capture the learnings from this field from the last two decades of research done in contemporary health systems in low and middle-income countries. We also used the snowball sampling approach (by referring to bibliographies of shortlisted publications) to identify additional publications related to scale-up. Two authors (SK, PDS) then screened the identified publications independently and relevant publications that discussed the attributes for a conceptual model for scale-up were shortlisted. We then included those publications that discussed attributes of scale up frameworks for public health interventions or innovations in low and middle-income countries for further analysis.
2. Further, we compared the strategies adopted in Rajasthan that led to rapid scale up within a context of available evidence on effectiveness with those described in the shortlisted frameworks to come up with a list of drivers and accelerators that support rapid scale-up of successful innovations in a time sensitive manner in LMIC settings. To do this, we obtained relevant project documents from the SCC program and reports and drew on authors’ implementation experiences and learning.

## Results

Through literature review, we identified 11 scale-up frameworks that fitted our search criteria. From these, we selected six (Table 1) that contained common themes, were contemporary (published after 1995, covering the last two decades), and that we felt reflected the current state of art thinking on scale-up theory and practice. These frameworks propose a sequential series of steps that support testing the innovation in the real world (i.e. within current health system, social and political context) and once proven impactful, to making it scale ready. They also identify several common themes influencing the success of scale-up, such as having a simple and context appropriate innovation; engaging important stakeholders such as the government and communities; using a feedback-based learning approach for testing; integrating innovations within the systems; ongoing advocacy for using data; and building system’s capacity to take over the innovations. Apart from a few side mentions, none of these frameworks explicitly calls out the need for or identifies drivers for expediting scale-ups. Spicer describes the attributes of innovations that support rapid diffusion--simplicity, low cost, compatibility and adaptability [9]. Both Spicer and Subramaniam highlight that scale-up requires medium to long-term timeframes, and make the case for longer donor funding cycles and commitment for testing, advocating for adoption, readiness for scale, and scale-up [8, 9]. Barker’s framework suggest it can take up to 6 years to go from developing ascendable units to at-scale implementation. Though the Barker framework sets out a sequential process of scaling up, it does acknowledge that streams of work can be initiated at different times and progress at different rates [23]. However, it does not explicitly identify this as a time saving factor.

**Table 1:** Overview of existing scale up frameworks

Conceptual models for scale-up	Major constructs influencing scale up	Reference to time sensitivity
Yamey G (2011)	Identifies following factors • Choose a simple intervention that is considered valuable. • Develop strong leadership • Ensure active engagement of range of stakeholders, including the target community • Incorporate research into implementation	Identifies 5 factors associated with faster diffusion of an innovation: • Relative advantage • Compatibility • Simplicity • Trialability • Observability Plus, • Integrate into existing health systems • Generation of timely evidence. • Timely feedback of monitoring data to implementers
Spicer N, et al. (2014)	Identifies multiple steps to catalyse scale up. • Design scalable innovations • Embed scale up in programme design • Build implementer capacity • Advocate on an on-going basis • Generate strong evidence • Involve government throughout project • Invoke policy champions and network of allies • Align with policy and targets • Promote community acceptance and uptake.	• Stresses the need for longer donor timelines and commitment for scale up, as it takes time for programmes to mature and for implementers to advocate and support government. Typical 2–3 year donor funding cycles are too short.
Subramanium S, et al. (2011)	• Tailor scale-up to fit the particular context. • Adopt a “learning by doing” approach, linking knowledge building with action. • Take into consideration the political, social, and economic environment. • Forge strong partnerships and adopt a participatory approach to foster ownership and sustainability • Focus on problem solving, and draw on a variety of quantitative and qualitative monitoring and evaluation methods. Don’t over rely on the randomised control trial approach.	• Acknowledges that successful scale up of pilot projects requires a medium to long term timeframe. • Slower, phased implementation, usually from the bottom up, which allows for systematic learning to emerge through incremental expansion based on concurrent, participatory research and adaptation
WHO ExpandNet: 1. Beginning with the end in mind, Planning Pilot Projects and other Programmatic Research for Successful Scale Up (2011) 2. Nine Steps for developing a Scaling Up Strategy (2010)	• Adopt a participatory process for innovation testing (including stakeholder involvement in the design, regular provision of feedback on implementation, nurture policy champions and wider networks). • Reach consensus on scaling up expectations • Tailor innovation to prevailing socio-cultural and institutional environment and test innovation under routine operating conditions and within existing resource constraints of the health system • Test ways to strengthen health-systems capacity as part of the project, for example (human or technical) • Advocate to donors and other funding sources for financial support for scale up once innovation proven to have impact • Search and test for sustainable finance • Prepare to advocate for necessary changes in policy regulation, and other health system context. • Promote learning and disseminate information. • Increase the capacity of the user organization to implement scale up. • Increase the capacity of the user team to support scaling up process • Prove support to vertical (institutionalization) and horizontal scale up (expansion/replication) scale up.	• No explicit reference to rapidity but Includes recommendation on advocacy with donors and other funding sources for financial support for scale up once innovation is proven to be successful. This will potentially expedite scale-up. Similarly, participatory process for innovation testing will potentially lead to rapid adoption.
Barker P. M, et al. (2016)	• Identifies 4 step sequential scale up process: o Set up (prepare ground and test intervention) o Develop scalable unit o Test scale up in different settings o Go to full scale • Enabling factors include: o Develop and engage leaders in their key role of guiding and supporting large scale change. o Accommodate context into design by starting with a deep dive situational analysis with key stakeholders. o Maintain a culture of urgency and persistence, and will to stay the course in proving an innovation and bringing it to scale. o Adoption of a learning approach which includes the continuous feedback of data to identify and close gaps in performance. o Communicate real time data and results on a regular basis.	• Although a linear scale up process is presented, the paper highlights it can be organic and iterative, with streams of work initiated at different times and progressing at different rates. Though not explicitly stated, this would be time saving. • Stresses rapid scale up will not occur in an unreceptive environment. • Country case studies suggest it can take up to 6 years to go from develop scalable unit to full scale.
Paina L and Peter D, (2012)	• Examines characteristics of scale up using an alternative model drawing on an understanding of complex adaptive systems (CAS): o Scale up occurs within complex and dynamic health systems, and the lens of CAS allows for better planning, implementation, monitoring and evaluation of scale up. o Lessons from CAS suggest giving more attention to local context, incentives, institutions, and paying greater attention to unintended consequences undermining scale up. o Includes adopting an approach that engages key stakeholders, through the transparent use of data on an on-going problem solving and adaption.	• CAS is a slow and deliberate approach. However, it recognises that a small stimulus can create a large or rapid change. • Phase transitions or tipping points can lead to rapid scale up. Acknowledges the importance of identifying the conditions under which rapid transitions can occur.

**Fig. 1:**
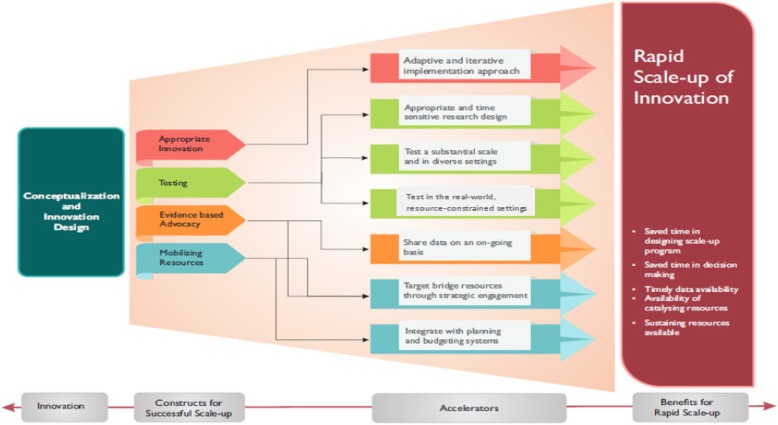
Accelerators for rapid scale-up of innovation

Comparing the constructs for successful at-scale implementation presented in these frameworks and drawing upon the experience of national scale-up of SCC programme, we identified the following seven ‘accelerators’ that significantly influence the rapidity of a scale-up (Fig. 1).

## Discussion

### Testing in the real-world, resource-constrained settings

One of the main tenets of the programme was to test SCC within the reality of a resource-constrained environment, using mostly the system’s resources to ensure that the incremental resource requirement was marginal and the system should be able to take these over in case the intervention is successful. This intentional effort was important from the perspective of scalability as there is a tendency for the pilots to be resource intensive to achieve results without giving due consideration to the system readiness. Only one framework, WHO Expandnet, recommends testing the innovation within routine operating conditions and existing resource constraints of health systems [11]. Spicer lists attributes of scalable health innovations such as being aligned and harmonized with the existing systems and priorities [9]. Yamey describes the importance of tailoring scale-up to the local situation and decentralizing delivery [10]. Yamey also touches upon research context for scale-up though it focuses mainly on systematic use of evidence for scale-up and the importance of synchronicity between implementation and research [10]. Caffe warns against setting up boutique projects, that are resource intensive and fail to be adopted by the government even if proven effective [24]. The need for testing of the innovation in real-world, resource-constrained settings needs to be deeply understood and elaborated for the public health community to realize the importance of achieving time-sensitive scale-up of interventions.

While the WHO SCC tool was designed to be used in most clinical settings [12, 20], the implementation team knew it was imperative to test it within the ‘real-world’ Indian health system to ensure successful scale-up by the government. For this reason, many intentional efforts were made in the SCC pilot in Rajasthan to keep it as simple and resource-light as possible. For example, the needed resources for training health providers on use of the WHO SCC and for ensuring availability of necessary drugs and equipment at the facility were largely sourced from within the health system. The orientation of health care providers on the use of SCC was restricted to just one and half days, and was undertaken at local training sites. This caused minimal disruption to the already staff crunched health system, a factor which has been seen as a challenge for scaling-up training programs [25]. Additionally, the trainers were selected from the existing pool of government trainers ensuring scalable training models.

We consider that choosing the setting in which an innovation is tested is critical in determining the speed of scale-up as it reduces the time otherwise taken up in modifying and adapting the innovation post-pilot to fit into system’s context. In addition, a resource light approach means minimal requirement of additional time for fund raising and securing supply systems. Alignment with existing national policies and guidelines saves time and eliminates the need to advocate for and support development of new policies and strategies.

### Appropriate and time sensitive research design

The existing frameworks emphasize on the need to generate robust evidence on intervention impact to inform scale-up. However, they do not provide guidance on the type of evaluation that are appropriate or on the importance of generating evidence in a timely manner for public health innovations. Spicer explores in detail the attributes of evidence for catalysing scale-up, but mostly from a presentation of information perspective. His framework lists various types of evidences such as quantitative evidence on outcomes and impact, cost-effectiveness, qualitative process data, mapping and needs assessment [9]. Subramaniam recommends a “learning by doing approach” and Spicer reports implementers favouring approaches with “flexibility to redesign and learn-do cycling” for successful scale-up [8, 9]. None of the frameworks recommend any hierarchy of the type of effectiveness measure that should trigger scale-up, i.e. should it always be at the level of impact on lives saved or can it occur on the basis of intermediate measures of effectiveness such as improved adherence to evidence-based practices.

Further, there has been recent acknowledgment in the literature that randomised control trials (RCTs) are not always the most appropriate method for testing an intervention [9, 26]. While they may be the gold standard approach for testing the efficacy of a drug or vaccine, they are not appropriate for proving effectiveness of an intervention embedded within a complex environment. Quasi-experimental methods – preferably, supplemented with qualitative methods may be more relevant as they shed light on ‘how’ and ‘how much’ the intervention has achieved impact [26]. Specifically, studies with quasi-experimental design that use control groups and pre-tests are considered adequate for evaluating interventions implemented in community settings [27]. Barker recommends testing of necessary infrastructure required for full-scale implementation and building capability of the system as an integral part of the testing phase itself [23]. An implementation research design allows for better understanding of these factors, whereas the gold standards tend to adjust or nullify the effects of these to ensure comparability among the study groups. For this reason, for the SCC pilot, we selected a quasi-experimental impact evaluation. This was supplemented with routine monitoring data, health provider interviews, an observational study on adherence to clinical practices in intervention and control sites and a cost effectiveness analysis.

Once the positive results of SCC on adherence to practices became available, and it was apparent the SCC was having a wider impact on quality of childbirth care [14], the GoI was keen to proceed with national implementation. Intermediate results on practice related outcomes were thought to be adequate to trigger scale-up as many of the key practices (such as newborn resuscitation with a bag and mask, and use of magnesium sulphate for management of pre-eclampsia and eclampsia) in the SCC have been independently proven to be lifesaving through independent studies [28–31].

Three aspects are worth highlighting in the manner in which the evidence generated through the SCC programme facilitated rapid scale-up. First, the decision to opt for a quasi-experimental evaluation design over an RCT saved considerable time and permitted mid-course adaptations. Second, as a result of the study design, important information was obtained regarding the system’s capacity to implement the intervention and required resource planning for scaling it up during the pilot phase itself. Third, GoI considered the intermediate results on improvement in adherence to key practices sufficient for decision-making. By the time an 11% perinatal mortality reduction was confirmed [15], the national scale-up was under planning.

### Testing at substantial scale and in diverse settings

There is often a tendency to undertake pilot studies for innovative interventions at a small scale, in controlled, and not-so diverse settings. This limits the applicability of the evidence generated from the study for scale-up in diverse settings. The SCC was tested in over hundred facilities located in seven districts of Rajasthan, catering to a population of about fifteen million. Additionally, the SCC was implemented at two levels of the health facilities--District Hospital and Community Health Centre—where most of the deliveries are conducted in Indian settings.

The ExpandNet framework refers to the limitations of conducting small scale, resource intensive pilots. While they may deliver positive impact, they are usually context specific and often fail to deliver results when taken to scale in different settings [11]. Barker stresses the importance of testing an intervention at scale, including different levels of the health system [23]. As the SCC was tested in a diverse range of health facilities and settings, there was confidence that it would work across the country. Furthermore, selection of the state was crucial from the perspective of extrapolation of evidence. Rajasthan is among the Empowered Action Group (EAG) states with weak health systems and indicators [32]. Thus, It could be inferred that, if SCC implementation was possible there, it will most likely work in other states of the country as well. Through this approach, there was considerable timesaving as the need for further testing in multiple settings and contexts after the successful pilot was eliminated.

### Adaptive and iterative prototyping approach to implementation

Almost all scale-up frameworks speak of the need to adopt an adaptive design approach that is to “learn while doing” and undertake modifications based on the emerging evidence. This iterative prototyping approach requires investments to be made in the generation of good-quality real-time monitoring data as well as special studies during the course of programme. This approach also sheds light on the “how” an innovation has been impactful, especially on the pathways of scaling up [33].

Through the course of implementation, the learning and adaptive approach used in the SCC programme showed that SCC is much more than a memory tool as originally envisioned. It provides a framework of action for multiple dimensions of quality of childbirth care such as improving resource availability, better supervision, and developing health worker competency building programs. Improved accountability as a result of the SCC being made an integral part of the client case-sheet was a new learning for the project implementers. This increased the SCC’s appeal to GoI, and their willingness to take it up on a national scale. Similarly, through the implementation, it was realized that there was a need for some on-site support to help the providers in institutionalizing the use of the SCC. Acting on this need, the SCC program sourced additional technical assistance to provide onsite support to the health providers to institutionalize SCC’s its use as a routine part of childbirth care. However, care was taken to ensure that this supportive supervision was minimal and something that the government could take up if they decided to go for scale-up. This was in line with earlier evidence where supportive supervision was reported to be a successful strategy for improving programme outcomes and health workers’ capacities [34, 35].

These efforts to iterate and prototype the interventions as well as the theory of change through the rigorous use of data helped the SCC to be scale ready by the time the implementation research was over, thereby improving the rapidity of scale-up.

### Sharing data and evidence on an ongoing basis

All frameworks report that sharing evidence in an ongoing and effective manner is critical for successful scale-up of an innovation. Spicer also reports that “well-presented information makes a humungous impact” and that apart from externally funded quantitative surveys on outcomes and impacts, other types of evidence including qualitative process data, first- hand experience and gaps assessments information is also important for scale-up. “Early and ongoing advocacy” was another factor reported as important for scale-up [9]. Barker also specifically reports the importance of real-time data as a powerful influencer of buy-in for scale-up [23].

The SCC programme included a full-scale of programme monitoring and evaluation activities that generated data on a periodic basis from all the available systems - reporting by facilities, monitoring and observation data reported by program officers, and specially designed studies for assessing adherence to safe care practices. This data was shared on a periodic basis with relevant stakeholders. Clinical leaders, programme managers, and policy makers were kept informed of the progress. Consistent sharing of data primed the government stakeholders in owning evidence and acting on it swiftly. This cut out the additional step of having to advocate separately for scale-up once the intervention has been shown to be impactful.

### Target bridge resources through strategic engagement of stakeholders

Most frameworks stress on the importance of engaging stakeholders throughout the programme implementation cycle for improving ownership and chances for scale-up. Yamey emphasises engagement of local implementers and other technical partners and using both state and non-state actors as implementers [10]. Both Barker and Yamey report the importance of local leadership for scale-up of successful innovations [10, 23]. However, apart from the importance of engaging local government and non-government implementation actors, we learned that engaging donors throughout the implementation process was one major factor influencing the speed of subsequent scale-up, should the pilot show positive results. The WHO Expandnet framework recognises the importance of early donor engagement to secure this funding [11].

In the SCC program, data was regularly shared with government authorities and donors. Multiple learning and exposure visits to programme sites by government policy makers and various donor representatives were organized to give them first-hand experience of the programme. In addition, GoI also kept these donor agencies engaged. This stakeholder engagement was instrumental in building GoI’s confidence in rapidly scaling up the innovation. Additional donor funding played an important catalytic role in kick-start the programme after its launch while government’s own funding was being secured. Due to the early and regular engagement with the donor agencies, securing additional donor funds for supporting this national initiative was not difficult.

### Timely integration of scale-up plans with the annual planning and budgeting cycles and systems

Many of the scale up frameworks refer to the need to align the proven innovation to existing polices, planning, and budgeting systems to support national uptake and expansion. These are key considerations in the process of institutionalising a successful innovation. An important aspect of institutionalisation is securing adequate and sustainable funding for scale up. This remains a significant challenge to scale-up, as most innovations have to compete for financial resources with the existing programs [8, 9]. This is an unexplored area in current scale up frameworks.

One way of overcoming this challenge is by ensuring the innovation is integral to current delivery mechanisms, and able to tap into existing funding streams, rather than requiring additional funds. Further, alignment of timelines of the pilot activities, such as data sharing and advocacy, to government’s planning and budgeting cycles can also improve the availability of resources at the time of scale-up thereby reducing the time-lag between the emergence of the evidence and subsequent scale-up.

Once the SCC was showing promising results on provider behaviour, the programme team supported the state government teams to reflect the SCC programme in the Rajasthan government’s annual national health mission budget in a timely manner. As a result, the new national quality improvement programme, centred around the SCC, was able to tap additional funds in time for scale-up. Moreover, as the SCC was well integrated into the existing maternal and newborn health programme, in reality, this did not require new funds, but a reallocation of funds within budget items in most cases. For example, the scale-up programme was able to utilise the existing training budget requested by the states for conducting refresher trainings.

These seven accelerators identified through our SCC pilot experience are important additions to the existing scale-up frameworks. These new insights can serve as a complement to current frameworks and provide valuable guidance to implementers for improving the rapidity of future scale-up initiatives in India and elsewhere.

### Strengths and limitations

This study is first of its kind and identifies and describes key accelerators to scaling up proven innovations within the health systems of LMICs. It draws upon the recent experience of authors in scaling up the SCC program in India – which has been able to harmonize scientific rigour with political urgency. The authors have not come across any literature which attempts to understand the nuances of ensuring time sensitive scale up of effective interventions which can avert countless deaths by minimizing the time lag between pilot and scale-up. Coming from a typical resource constrained setting, the findings are applicable to multiple such settings across low and middle income countries.

This study has a few limitations. The review of literature was limited to published and grey literature in English language only; relevant publications in other languages were not included. Also, shortlisting of relevant scale-up frameworks from published literature was based on authors’ assessment and not on any objective criteria. However, since this was not supposed to be a systematic literature review, this was a conscious decision by the authors.

## Conclusion

The SCC programme demonstrates that it is possible to harmonise public health and political urgency without compromising on scientific rigour. The SCC programme’s experience illustrates how adherence to the main principles laid out in the scale-up frameworks such as choosing an appropriate intervention, testing the intervention rigorously, engaging important stakeholders through evidence-based advocacy, and mobilization of resources for scale-up lead to innovation uptake and expansion. However, the SCC programme provides important additional lessons on how to minimise the time lag between proving an intervention and its scale up. We recommend seven main accelerators for rapid scale-up of proven interventions.

The most important accelerator is the selection of the most appropriate, time sensitive and robust research design. Most impact evaluations have adopted RCT as the gold standard methodology. However, the long duration combined with limited transferability of proven innovations (due to the context specific nature of success) has led many to recently question this methodology. The SCC experience endorses this viewpoint. A related point, that has not received much attention, is what type of impact measure should trigger scale-up. Should it always be related to mortality reduction or are intermediate measures such as behaviour change adequate? Again, the SCC experience would suggest provider behaviour change is adequate if these changes themselves are recognized as effective in reducing mortality or morbidity.

While most frameworks speak of a sequential series of steps (pilot test, advocate, scale-up, etc.), the SCC programme illustrates that it is possible to initiate these streams of work in parallel and save considerable time. For example, the groundwork for scale-up was initiated well before the end of the pilot, based on the evidence on provider behaviour change. The national initiative guidelines were written during the third year of the pilot. Being flexible and responsive to government demands was finely balanced with the need to protect the integrity of evaluation. Actual scale-up only commenced once data collection for pilot’s evaluation was over.

We urge that these seven accelerators be incorporated into the scale-up frameworks, to guide future testing and scale-up in a timely manner. In light of the huge remaining burden of preventable maternal and newborn deaths, it is imperative that more attention is given to how progress can be made quickly. The SCC programme provides important lessons and guidance on how this can be achieved.

## Abbreviations

GoI: Government of India
GoR: Government of Rajasthan
LMIC: Low- and middle income countries
MDG: Millennium Development Goals
RCT: Randomised control trials
SCC: Safe Childbirth checklist
